# The role of GLS1-mediated glutaminolysis/2-HG/H3K4me3 and GSH/ROS signals in Th17 responses counteracted by PPARγ agonists

**DOI:** 10.7150/thno.54803

**Published:** 2021-03-04

**Authors:** Yumeng Miao, Yun Zheng, Yanzhi Geng, Ling Yang, Na Cao, Yue Dai, Zhifeng Wei

**Affiliations:** Department of Pharmacology of Chinese Materia Medica, School of Traditional Chinese Pharmacy, China Pharmaceutical University, 24 Tong Jia Xiang, Nanjing 210009, China.

**Keywords:** PPARγ, Th17 responses, glutaminolysis, glutaminase 1

## Abstract

**Background:** Peroxisome proliferator-activated receptor gamma (PPARγ) has the ability to counter Th17 responses, but the full mechanisms remain elusive. Herein, we aimed to elucidate this process in view of cellular metabolism, especially glutaminolysis.

**Methods:** MTT, CCK-8, Annexin V-FITC/PI staining or trypan blue exclusion assays were used to analyze cytotoxicity. Flow cytometry and Q-PCR assays were applied to determine Th17 responses. The detection of metabolite levels using commercial kits and rate-limiting enzyme expression using western blotting assays was performed to illustrate the metabolic activity. ChIP assays were used to examine H3K4me3 modifications. Mouse models of dextran sulfate sodium (DSS)-induced colitis and house dust mite (HDM)/lipopolysaccharide (LPS)-induced asthma were established to confirm the mechanisms studied *in vitro*.

**Results:** The PPARγ agonists rosiglitazone and pioglitazone blocked glutaminolysis but not glycolysis under Th17-skewing conditions, as indicated by the detection of intracellular lactate and α-KG and the fluorescence ratios of BCECF-AM. The PPARγ agonists prevented the utilization of glutamine and thus directly limited Th17 responses even when Foxp3 was deficient. The mechanisms were ascribed to restricted conversion of glutamine to glutamate by reducing the expression of the rate-limiting enzyme GLS1, which was confirmed by GLS1 overexpression. Replenishment of α-KG and 2-HG but not succinate weakened the effects of PPARγ agonists, and α-KG-promoted Th17 responses were dampened by siIDH1/2. Inhibition of KDM5 but not KDM4/6 restrained the inhibitory effect of PPARγ agonists on IL-17A expression, and the H3K4me3 level in the promoter and CNS2 region of the *il-17* gene locus down-regulated by PPARγ agonists was rescued by 2-HG and GLS1 overexpression. However, the limitation of PPARγ agonists on the mRNA expression of RORγt was unable to be stopped by 2-HG but was attributed to GSH/ROS signals subsequent to GLS1. The exact role of PPARγ was proved by GW9662 or PPARγ knockout, and the mechanisms for PPARγ-inhibited Th17 responses were further confirmed by GLS1 overexpression* in vivo*.

**Conclusion:** PPARγ agonists repressed Th17 responses by counteracting GLS1-mediated glutaminolysis/2-HG/H3K4me3 and GSH/ROS signals, which is beneficial for Th17 cell-related immune dysregulation.

## Introduction

CD4^+^ T cells are the main component of the adaptive immune system. Upon stimulation, they activate, proliferate, and differentiate into different functional subsets, including T helper (Th) 1, Th2, Th17, T follicular helper (Tfh) and regulatory T (Treg) cells 1. Since its discovery in 2005, the Th17 class of cells has attracted intensive research endeavors 1. These IL-17A-, IL-21- and IL-22- producing cells, which are polarized under the control of retinoic acid receptor-related orphan receptor γt (RORγt), have been well confirmed to be indulged in promoting inflammation and autoimmune phenomena apart from immune defenses against infections 1,2. For example, in an adoptive transfer model of colitis, transferring CD4^+^CD25^-^ T cells from RORγt^-/-^ mice to RAG-1^-/-^ immunodeficient mice is unable to induce colitis, while treatment with IL-17A can restore colitis after the transfer of RORγt^-/-^ T cells 3. Moreover, neutralization of IL-17A is sufficient to inhibit house dust mite (HDM)-induced asthma in mice 4. Therefore, modulating desired T-cell responses by targeting Th17 cells has clinical potential in the treatment of relevant diseases such as colitis and asthma 3-5.

Peroxisome proliferator-activated receptor gamma (PPARγ) is a transcription factor that can form heterodimers with retinoid X receptors (RXRs) and regulate gene transcription in a ligand-dependent or ligand-independent manner 6. The receptor has long been considered the target protein of thiazolidinediones, a class of anti-diabetic drugs, and is involved in the regulation of insulin resistance 7. Of note, a growing body of evidence indicates that PPARγ also plays an important role in Th17 responses. When compared with wild-type CD4^+^ T cells, T cell-specific knockout of PPARγ leads to stronger Th17 differentiation. In addition, studies on PPARγ activated by endogenous ligands or synthetic agonists have further confirmed it to be a repressor of Th17 cells 8,9. However, the in-depth mechanisms of PPARγ as a promising molecular target for specific intervention in Th17 responses are still in doubt and need more investigation.

Recently, an expanding spectrum of research efforts has extended the concept that metabolism and immune cells are linked, and the significance of metabolic processes in Th17 responses has been substantiated by genetic methods or using inhibitors of rate-limiting enzymes 10. It is worth noting that several published papers have revealed the relationship between PPARγ and glutaminolysis. When PPARγ is activated, the reduced conversion rate of glutamine to glutamate results in restricted proliferation of lung cancer cells 11. Furthermore, phosphatase and tensin homologue deleted on chromosome ten (PTEN), the downstream target gene of PPARγ, mediates the ubiquitination-proteasome degradation of glutaminase 1 (GLS1), the enzyme in the first step of glutaminolysis 12. Thus, in this study, we explored whether PPARγ could inhibit Th17 responses through the regulation of cellular metabolism, especially glutaminolysis, and further uncovered the subsequent mechanisms.

## Results

### PPARγ agonists inhibit glutaminolysis rather than glycolysis under Th17-skewing conditions

**Figure 1:**
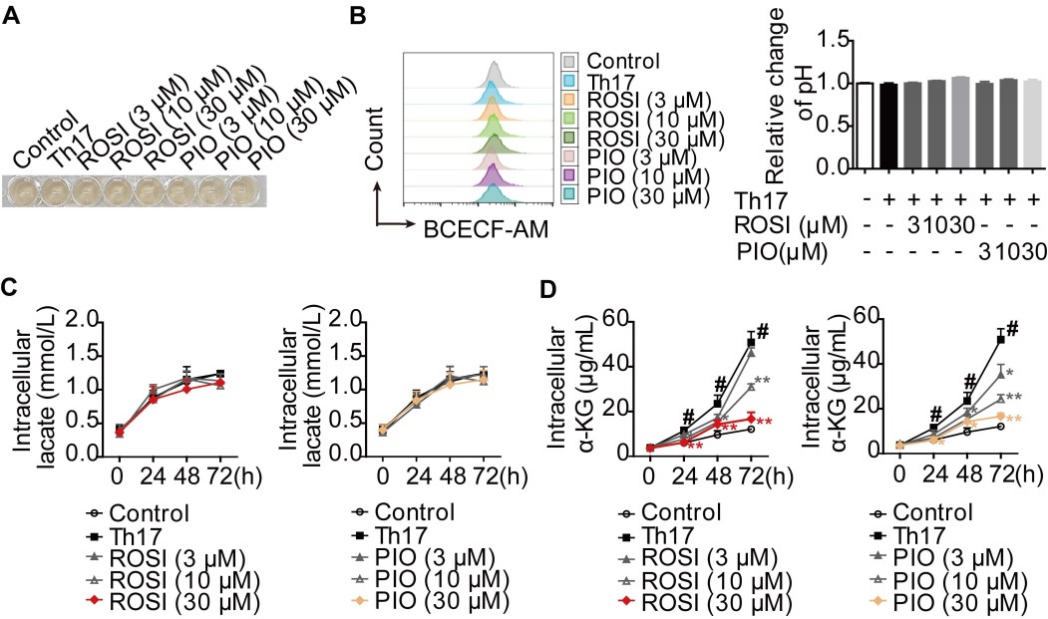
** PPARγ agonists inhibit glutaminolysis rather than glycolysis under Th17-skewing conditions.** (A-D) The naïve CD4^+^ T cells were prepared, and treated with anti-CD3/CD28 in the presence or absence of Th17-skewing cytokines, rosiglitazone (ROSI; 3, 10, 30 µM) as well as pioglitazone (PIO; 3, 10, 30 µM). After 72 h, the visible changes of pH were showed by the color of cell culture media (A), and the relative intracellular pH values were determined by flow cytometry using fluorescence ratios of BCECF-AM between the green and orange channels (FL1/FL3) (B). At 0, 24, 48 and 72 h, the concentrations of intracellular lactate (C) and α-KG (D) were determined by using commercial kits. Data were presented as the means ± S.E.M. of three independent experiments. ^#^*P* < 0.05 vs. Control group or the group without any treatment; ^*^P < 0.05, ^**^P < 0.01 vs. Th17 group (Model group).

Th17 cells preferentially use glycolysis and glutaminolysis for their energy supply 13-15. Therefore, we evaluated the effects of PPARγ agonists on these two metabolic pathways under Th17-skewing conditions. First, the cytotoxicities of the tool drugs, rosiglitazone (ROSI; 0-100 μM) and pioglitazone (PIO; 0-100 μM), were examined by MTT, CCK-8 and Annexin V-FITC/PI assays. However, no significant effect could be seen in terms of cell viability or apoptosis (Figure S1A-B). Moreover, the changes in pH and lactate level were evaluated as indicators of glycolysis. When naïve CD4^+^ T cells were stimulated and cultured with TGF-β, IL-6 and IL-23, we found that ROSI (3, 10, 30 μM) and PIO (3, 10, 30 μM) altered neither the intracellular pH, which was positively correlated with the fluorescence ratios of BCECF-AM (a pH-sensitive dye) 16, nor the colors of the culture media (Figure 1A-B). The determination of intracellular lactate, as shown in Figure 1C, revealed no profound effect of ROSI (3, 10, 30 μM) or PIO (3, 10, 30 μM), further indicating their negligible impact on glycolysis under Th17 conditions. However, the level of α-ketoglutarate (α-KG), a product of glutaminolysis, was down-regulated by ROSI (10, 30 μM) and PIO (10, 30 μM) (Figure 1D). These results imply that PPARγ agonists mainly inhibit glutaminolysis rather than glycolysis under Th17-skewing conditions.

### PPARγ agonists restrict the utilization of glutamine and thus directly inhibit Th17 differentiation

**Figure 2:**
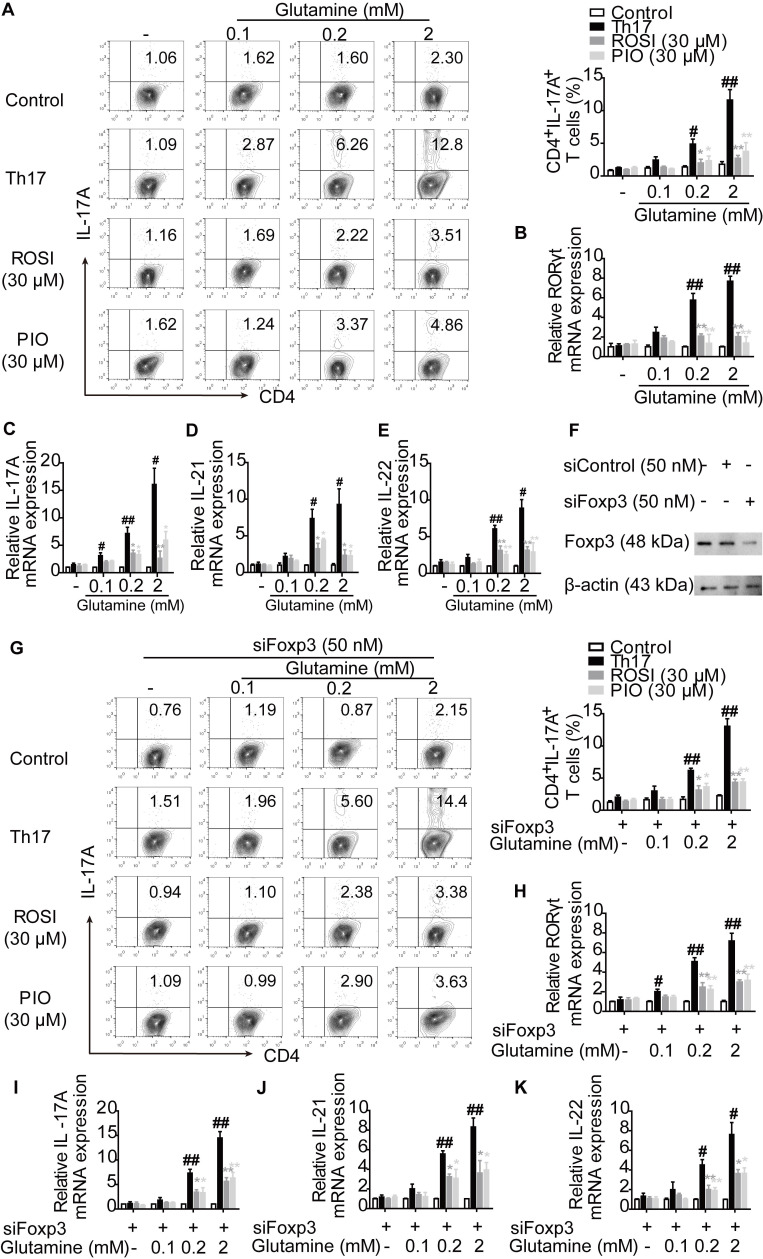
** PPARγ agonists prevent the utilization of glutamine and result in the limitation of Th17 differentiation directly.** (A-E) The naïve CD4^+^ T cells were prepared, and treated with anti-CD3/CD28 in the presence or absence of Th17-skewing cytokines, glutamine (0.1, 0.2, 2 mM), rosiglitazone (ROSI; 30 µM) as well as pioglitazone (PIO; 30 µM) for 72 h. Then, the frequency of CD4^+^IL-17A^+^ T cells was detected by flow cytometry (A), and the relative mRNA expression levels of RORγt, IL-17A, IL-21 as well as IL-22 were determined by Q-PCR assay (B-E). (F) The naïve CD4^+^ T cells were transfected with siFoxp3, and the protein expressions of Foxp3 was analyzed by western blotting assay. (G-K) The naïve CD4^+^ T cells were transfected with siFoxp3, and then treated with anti-CD3/CD28 in the presence or absence of Th17-skewing cytokines, glutamine (0.1, 0.2, 2 mM), ROSI (30 µM) as well as PIO (30 µM) for 72 h. The frequency of CD4^+^IL-17A^+^ T cells was detected by flow cytometry (G), and the relative mRNA expression levels of RORγt, IL-17A, IL-21 as well as IL-22 were determined by Q-PCR assay (H-K). Data were presented as the means ± S.E.M. of three independent experiments. ^#^*P* < 0.05, ^##^*P* < 0.01 vs. Control group; ^*^P < 0.05, ^**^P < 0.01 vs. Th17 group (Model group).

To further determine the role of glutaminolysis in PPARγ-mediated restriction under Th17-skewing conditions, glutamine was deprived from the cell culture. We found that the survival rates of lymphocytes were comparable between the groups cultured with or without glutamine, and no significant changes were observed by further adding ROSI (30 μM) and PIO (30 μM) (Figure S2). Corresponding to the published significance of glutaminolysis in Th17 responses 15, few IL-17A^+^ T cells were generated when no glutamine fueled the differentiation, and many more Th17 cells could be detected by re-adding the indicated concentrations of glutamine into the culture media (Figure 2A). However, the naïve CD4^+^ T cells co-cultured with ROSI (30 μM) and PIO (30 μM) were refractory to the induction of Th17 cells in the presence of glutamine, as the frequencies of polarized Th17 cells were distinctly lower than those without adding PPARγ agonists (Figure 2A). As shown in Figure 2B-E, the determination of the mRNA levels of the Th17-specific transcription factor RORγt and the functional cytokines IL-17A, IL-21 and IL-22 further confirmed the results, indicating that PPARγ agonists prevented the cells from utilizing or metabolizing glutamine and thus restricted the formation of Th17 cells. Considering that the shift of Th17 cells towards Treg cells might be the reason why PPARγ agonists reduced the frequencies of Th17 cells facilitated by glutamine, siFoxp3 was used, and the results in Figure 2F-K show that ROSI (30 μM) and PIO (30 μM) still abolished the effect of glutamine on Th17 differentiation in Foxp3-deficient cells. Thes e data reveal that PPARγ agonists can directly inhibit Th17 differentiation by restricting the utilization of glutamine.

### PPARγ agonists block GLS1-mediated glutaminolysis under Th17-skewing conditions

**Figure 3:**
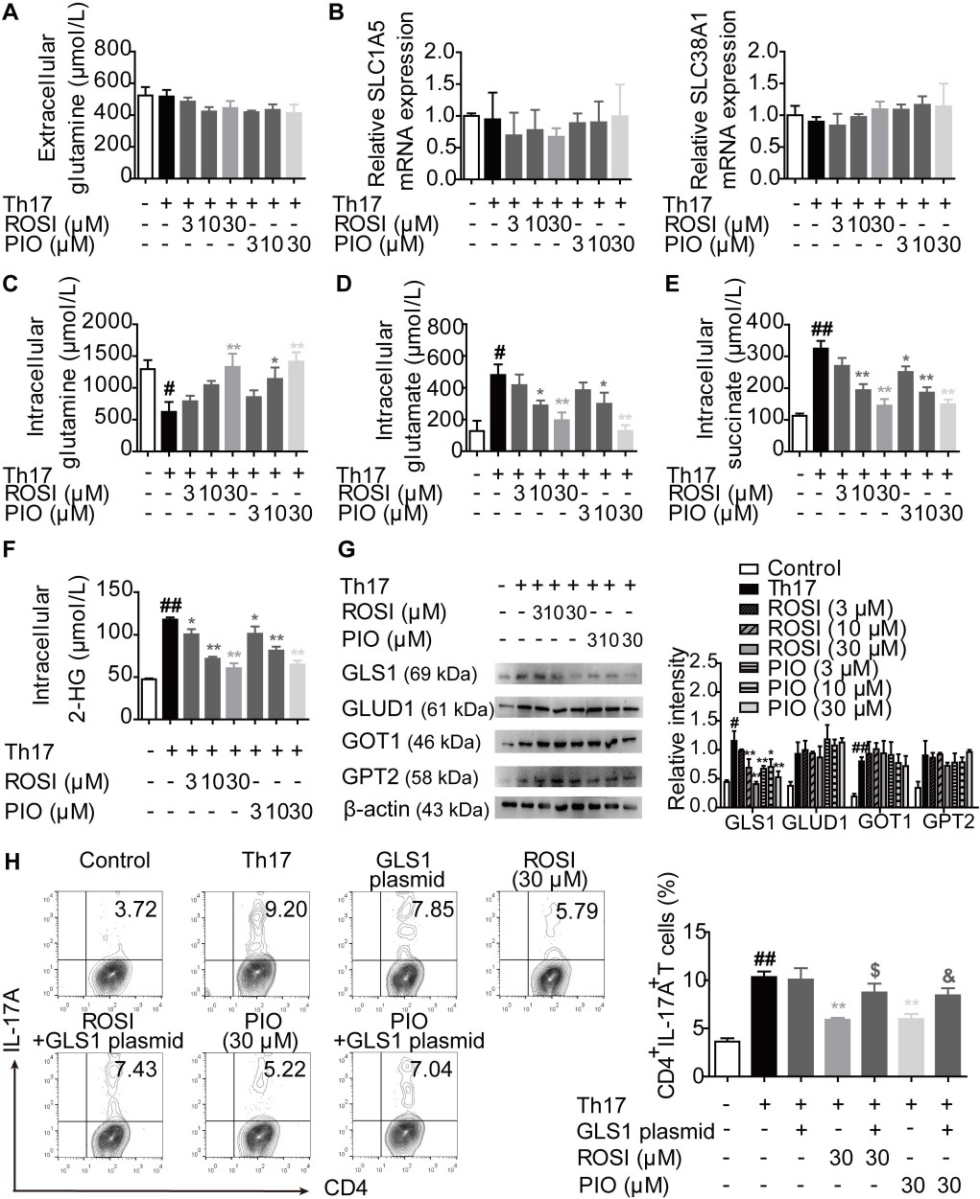
** PPARγ agonists block glutaminolysis *via* a restricted expression of GLS1.** The naïve CD4^+^ T cells were prepared, and treated with anti-CD3/CD28 in the presence or absence of Th17-skewing cytokines, rosiglitazone (ROSI; 3, 10, 30 µM) as well as pioglitazone (PIO; 3, 10, 30 µM) for 48 h. (A) The concentration of extracellular glutamine was detected by using a commercial kit. (B) The relative mRNA expression levels of SLC1A5 and SLC38A1 were examined by Q-PCR assay. (C-F) The concentrations of intracellular glutamine (C), glutamate (D), succinate (E) and 2-HG (F) were determined by using commercial kits. (G) The protein levels of GLS1, GLUD1, GOT1 and GPT2 were analyzed by western blotting assay. (H) The naïve CD4^+^ T cells were transfected with GLS1 plasmid, and then treated with anti-CD3/CD28, Th17-skewing cytokines, ROSI (30 µM) or PIO (30 µM) for 72 h. The frequency of CD4^+^IL-17A^+^ T cells was detected by flow cytometry. Data were presented as the means ± S.E.M. of three independent experiments. ^#^*P* < 0.05, ^##^*P* < 0.01 vs. Control group; ^*^P < 0.05, ^**^P < 0.01 vs. Th17 group (Model group); ^$^*P* < 0.05 vs. ROSI group; ^&^*P* < 0.05 vs. PIO group.

The process of glutaminolysis begins with the uptake of glutamine into the cytosol through amino acid transporters 17,18. Then, glutamine is deaminated to glutamate via GLS and subsequently converted to α-KG by glutamate dehydrogenase (GLUD), glutamic oxaloacetic transaminase (GOT) or glutamic pyruvic transaminase (GPT) 17,18. To determine whether PPARγ agonists restricted the uptake of glutamine, we detected the level of glutamine and the mRNA expression of the transporters SLC1A5 and SLC38A1 under Th17-skewing conditions. The results showed that ROSI (3, 10, 30 μM) and PIO (3, 10, 30 μM) exerted no obvious effect on the extracellular level of glutamine, implying that no additional glutamine was transported into the cytosol (Figure 3A). In addition, the mRNA expression levels of the transporters SLC1A5 and SLC38A1 were not affected by the PPARγ agonists (Figure 3B). However, as revealed in Figure 3C-F, the intracellular level of glutamine was up-regulated by ROSI and PIO in a concentration-dependent manner, while the levels of glutamate and α-KG, together with the downstream metabolites of α-KG such as succinate and 2-hydroxyglutarate (2-HG), were down-regulated, indicating an inhibitory effect of PPARγ agonists on the conversion of glutamine to glutamate. Furthermore, the protein level of the rate-limiting enzyme GLS1 but not GLUD1, GOT1, or GPT2 was consistently reduced by PPARγ agonists (Figure 3G), and transfection of the GLS1 overexpression plasmid reversed their limitation on the frequency of Th17 cells (Figure 3H). Thus, PPARγ agonists block GLS1-mediated glutaminolysis and subsequently restrict the generation of Th17 cells.

### PPARγ agonists-inhibited Th17 responses are mediated by the glutamine metabolite 2-HG

**Figure 4:**
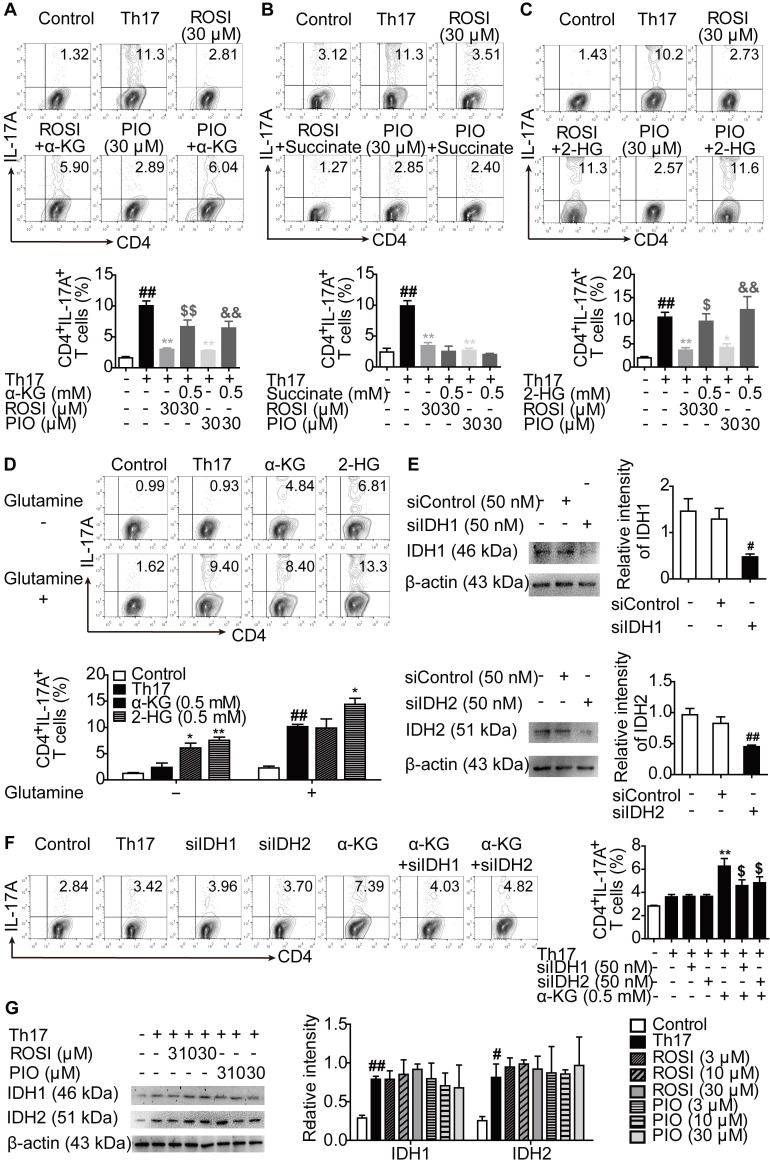
** 2-HG is involved in the inhibition of PPARγ agonists on Th17 differentiation.** (A-C) The naïve CD4^+^ T cells were prepared and treated with anti-CD3/CD28 in the presence or absence of Th17-skewing cytokines, α-KG (0.5 mM), succinate (0.5 mM), 2-HG (0.5 mM), rosiglitazone (ROSI; 30 µM) as well as pioglitazone (PIO; 30 µM) for 72 h, and the frequency of CD4^+^IL-17A^+^ T cells was detected by flow cytometry. (D) The naïve CD4^+^ T cells were treated with anti-CD3/CD28 in the presence or absence of Th17-skewing cytokines, glutamine (2 mM), α-KG (0.5 mM) as well as 2-HG (0.5 mM) for 72 h, and the frequency of CD4^+^IL-17A^+^ T cells was detected by flow cytometry. (E) The naïve CD4^+^ T cells were transfected with siIDH1 or siIDH2, and the protein levels of IDH1 and IDH2 were analyzed by western blotting assay. (F) The naïve CD4^+^ T cells were transfected with siIDH1 or siIDH2, and then treated with anti-CD3/CD28, Th17-skewing cytokines, α-KG (0.5 mM) in the cell culture without glutamine for 72 h. The frequency of CD4^+^IL-17A^+^ T cells was detected by flow cytometry. (G) The naïve CD4^+^ T cells were treated with anti-CD3/CD28 in the presence or absence of Th17-skewing cytokines, ROSI (3, 10, 30 µM) as well as PIO (3, 10, 30 µM) for 48 h. The protein levels of IDH1 and IDH2 were analyzed by western blotting assay. Data were presented as the means ± S.E.M. of three independent experiments. ^#^*P* < 0.05, ^##^*P* < 0.01 vs. Control group or the group without any treatment; ^*^P < 0.05, ^**^P < 0.01 vs. Th17 group (Model group); ^$^*P* < 0.05, ^$$^*P* < 0.01 vs. ROSI group or α-KG group; ^&^*P* < 0.05, ^&&^*P* < 0.01 vs. PIO group.

We further explored how PPARγ agonists interfered with Th17 differentiation after constraining glutaminolysis. As shown in Figure 4A, when supplemented with cell-permeable α-KG, the inhibition of Th17 differentiation by ROSI (30 μM) and PIO (30 μM) was attenuated. To determine whether α-KG itself or its downstream metabolites were involved, cell-permeable succinate and 2-HG were added to the cell culture, and 2-HG but not succinate exerted a similar effect as α-KG (Figure 4B-C). Interestingly, in glutamine-sufficient culture, only 2-HG further boosted the generation of Th17 cells, although supplementation with either α-KG or 2-HG resulted in more CD4^+^IL-17A^+^ T cells when glutamine was deprived (Figure 4D). Furthermore, isocitrate dehydrogenase 1 (IDH1) and IDH2 are the enzymes mediating the conversion of α-KG to 2-HG 19. By genetically knocking down IDH1 or IDH2 using effective small interfering RNAs (siRNAs), the promotion of α-KG on Th17 differentiation in glutamine-deprived culture was ablated (Figure 4E-F). In addition, ROSI (3, 10, 30 μM) and PIO (3, 10, 30 μM) had no effect on the protein expression of IDH1 or IDH2 (Figure 4G). These findings indicate that 2-HG is the functional effector of T cell specification and its response to PPARγ agonists.

### PPARγ agonists regulate KDM5-specific H3K4me3 modifications at the *il-17* gene locus owing to restriction of the GLS1/2-HG axis

**Figure 5:**
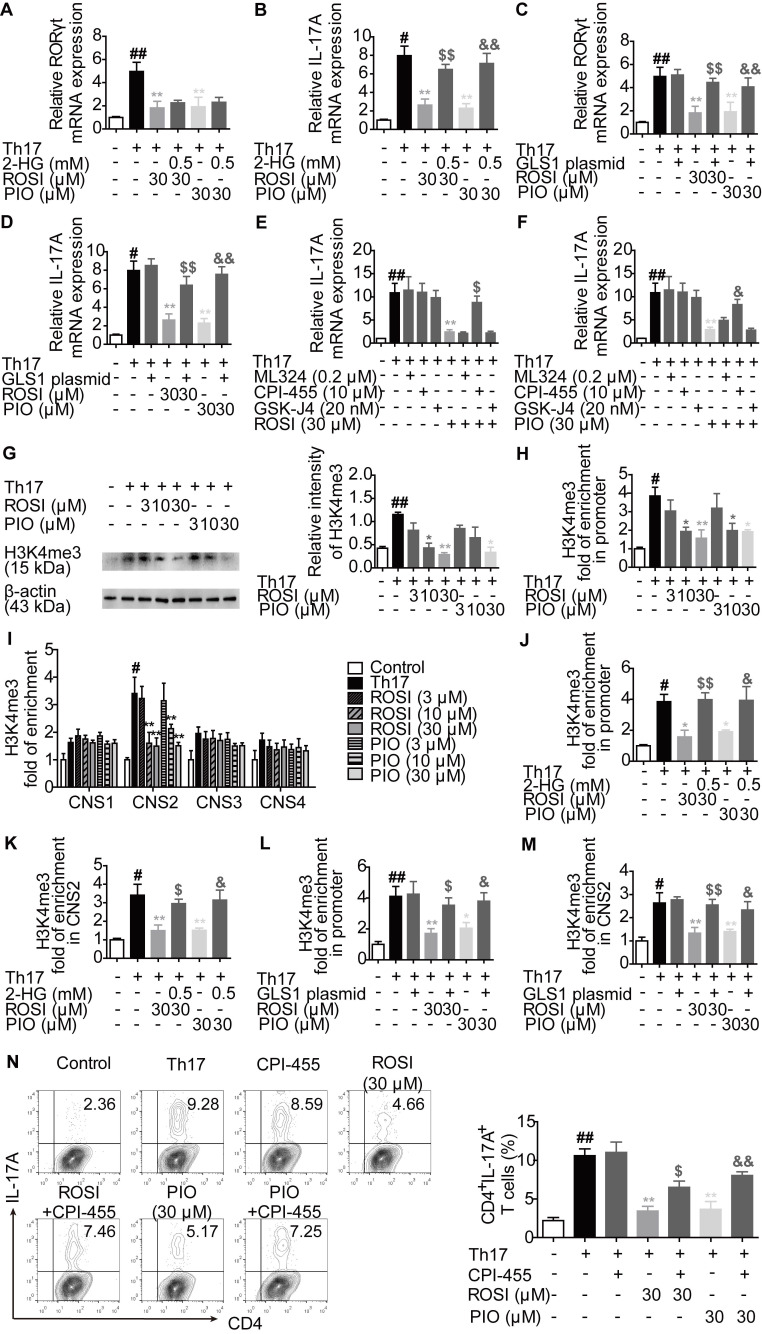
** PPARγ agonists limit the KDM5-specific H3K4me3 modifications in *il-17* gene locus through regulating the GLS1/2-HG axis.** (A-B) The naïve CD4^+^ T cells were prepared and treated with anti-CD3/CD28 in the presence or absence of Th17-skewing cytokines, 2-HG (0.5 mM), rosiglitazone (ROSI; 30 µM) as well as pioglitazone (PIO; 30 µM) for 72 h. The relative mRNA expression levels of RORγt (A) and IL-17A (B) were determined by Q-PCR assay. (C-D) The naïve CD4^+^ T cells were transfected with GLS1 plasmid, and then treated with anti-CD3/CD28, Th17-skewing cytokines, ROSI (30 µM) or PIO (30 µM) for 72 h. The relative mRNA expression levels of RORγt (C) and IL-17A (D) were determined by Q-PCR assay. (E-F) The naïve CD4^+^ T cells were treated with anti-CD3/CD28 in the presence or absence of Th17-skewing cytokines, ML324 (0.2 µM), CPI-455 (10 µM), GSK-J4 (20 nM), ROSI (30 µM) as well as PIO (30 µM) for 72 h. The relative mRNA expression of IL-17A was detected by Q-PCR. (G-I) The naïve CD4^+^ T cells were prepared, and treated with anti-CD3/CD28 in the presence or absence of Th17-skewing cytokines, ROSI (3, 10, 30 µM) as well as PIO (3, 10, 30 µM) for 72 h. The protein level of H3K4me3 was analyzed by western blotting assay (G), and the enrichment of H3K4me3 in promoter (H) and CNS1, 2, 3, 4 (I) region of* il-17* gene was analyzed by ChIP. (J-K) The naïve CD4^+^ T cells were treated with anti-CD3/CD28 in the presence or absence of Th17-skewing cytokines, 2-HG (0.5 mM), ROSI (30 µM) as well as PIO (30 µM) for 72 h. The enrichment of H3K4me3 in promoter (J) and CNS2 (K) region of* il-17* gene was analyzed by ChIP. (L-M) The naïve CD4^+^ T cells were transfected with GLS1 plasmid, and then treated with anti-CD3/CD28, Th17-skewing cytokines, ROSI (30 µM) or PIO (30 µM) for 72 h. The enrichment of H3K4me3 in promoter (L) and CNS2 (M) region of* il-17* gene was analyzed by ChIP. (N) The naïve CD4^+^ T cells were treated with anti-CD3/CD28 in the presence or absence of Th17-skewing cytokines, CPI-455 (10 µM), ROSI (30 µM) as well as PIO (30 µM) for 72 h. The frequency of CD4^+^IL-17A^+^ T cells was determined by flow cytometry. Data were presented as the means ± S.E.M. of three independent experiments. ^#^*P* < 0.05, ^##^*P* < 0.01 vs. Control group; ^*^P < 0.05, ^**^P < 0.01 vs. Th17 group (Model group); ^$^*P* < 0.05, ^$$^*P* < 0.01 vs. ROSI group; ^&^*P* < 0.05, ^&&^*P* < 0.01 vs. PIO group.

To further confirm that the GLS1/2-HG axis was involved in the effects of PPARγ agonists on Th17 differentiation, we measured whether PPARγ agonists-reduced GLS1 expression and 2-HG level had a link with the mRNA expression of RORγt and IL-17A. However, as shown in Figure 5A-D, although both the exogenous 2-HG and GLS1 overexpression plasmid rescued the inhibitory effect of ROSI (30 μM) and PIO (30 μM) on the mRNA expression of IL-17A, 2-HG was unable to combat the limitation of PPARγ agonists on the mRNA expression of RORγt, which was different from the GLS1 plasmid and indicated an additional role of GLS1 in RORγt-regulated IL-17A expression.

To explore the downstream mechanisms of 2-HG in IL-17A expression, the involvement of histone methylation was evaluated since the activating histone marks of H3K4 methylation (H3K4me3) are reported to be up-regulated in the locus of the* il-17* gene, which facilitate its transcription as well as the Th17 responses 20-22, and 2-HG is an inhibitor of JmjC domain-containing demethylases (JMJDs), a class of enzymes capable of regulating the level of histone methylation 23. As revealed in Figure 5E-F, CPI-455, an inhibitor of the specific H3K4 demethylase KDM5 belonging to JMJD, rather than ML324 (an inhibitor of H3K9 or H3K36 demethylase KDM4) and GSK-J4 (an inhibitor of H3K27 demethylase KDM6), reversed the limitation of ROSI (30 μM) and PIO (30 μM) on the mRNA expression of IL-17A. The protein level of H3K4me3 was consistently down-regulated by ROSI and PIO in a concentration-dependent manner (Figure 5G). In addition, both ROSI (10, 30 μM) and PIO (10, 30 μM) restricted the levels of H3K4me3 in the promoter and CNS2 but not in the CNS1, 3 and 4 regions of the *il-17* locus, which were rescued by 2-HG and GLS1 plasmid (Figure 5H-M). Moreover, the ROSI (30 μM) and PIO (30 μM)-reduced frequencies of CD4^+^IL-17A^+^ T cells were attenuated by CPI-455 (Figure 5N). Collectively, PPARγ agonists modulate KDM5-specific H3K4me3 modifications in the *il-17* gene locus by interfering with the GLS1/2-HG axis.

### PPARγ agonists inhibit RORγt expression by regulating GLS1/GSH/ROS signals

In addition to α-KG, glutamate is also a source of glutathione (GSH) de novo synthesis, a process regulated by the glutamate-cysteine ligase catalytic subunit (GCLC), the glutamate-cysteine ligase modifier subunit (GCLM) and the glutathione synthase (GS) 24. The intervention in GLS1-mediated glutaminolysis, which reduces the level of glutamate, may result in the disturbance of the cellular GSH/reactive oxygen species (ROS) system 24,25. Therefore, we determined whether the additional effect of PPARγ agonists on GLS1-regulated RORγt expression was related to the GSH/ROS bypath.

**Figure 6:**
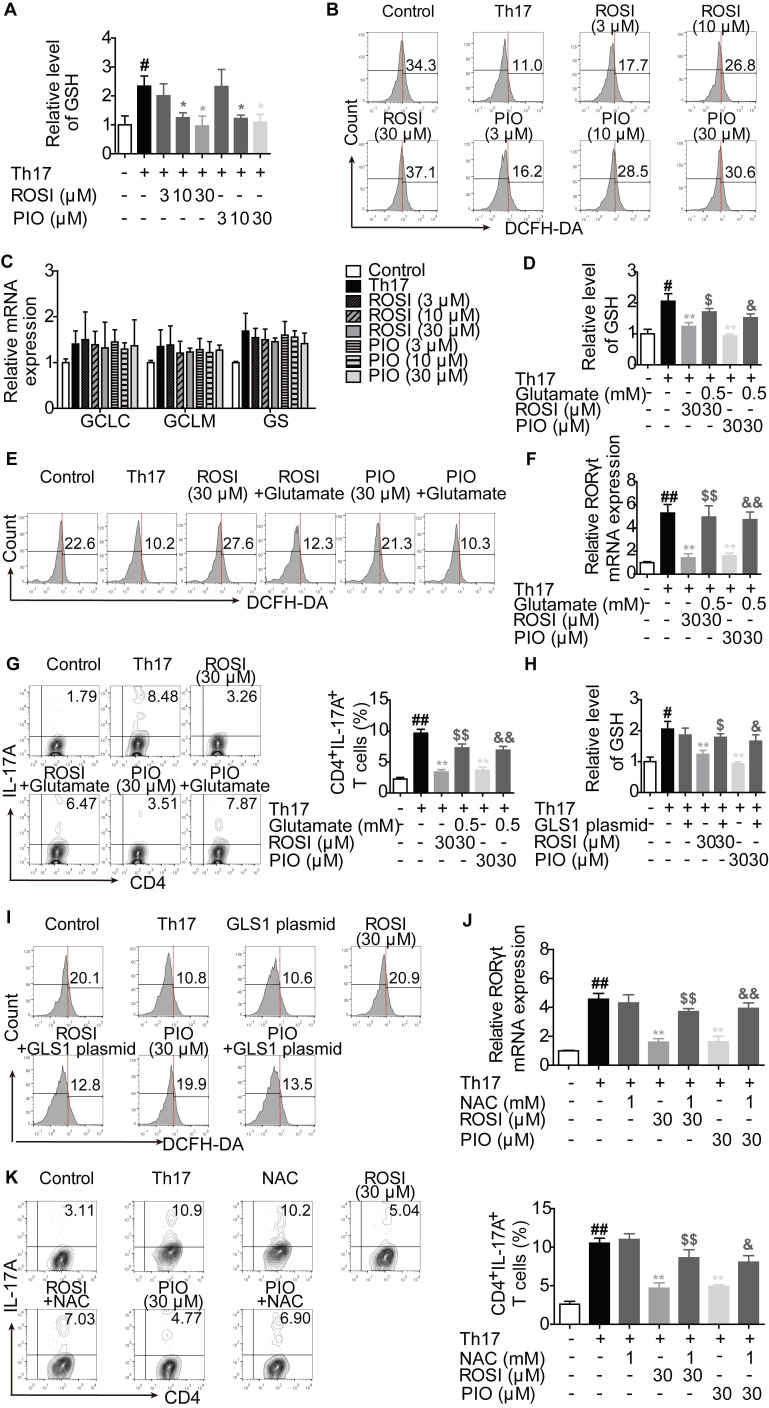
**PPARγ agonists inhibit RORγt expression *via* regulating GLS1/GSH/ROS signals.** (A-C) The naïve CD4^+^ T cells were prepared, and treated with anti-CD3/CD28 in the presence or absence of Th17-skewing cytokines, rosiglitazone (ROSI; 3, 10, 30 µM) as well as pioglitazone (PIO; 3, 10, 30 µM) for 48 h. The concentration of intracellular GSH was detected by using a commercial kit (A), the level of ROS was determined by flow cytometry (B), and the mRNA expression levels of GCLC, GCLM and GS were analyzed by Q-PCR (C). (D-G) The naïve CD4^+^ T cells were treated with anti-CD3/CD28 in the presence or absence of Th17-skewing cytokines, glutamate (0.5 mM), ROSI (30 µM) as well as PIO (30 µM). After 48 h, the concentration of intracellular GSH was detected by using a commercial kit (D), and the level of ROS was determined by flow cytometry (E). After 72 h, the mRNA expression of RORγt was detected by Q-PCR (F), and the frequency of CD4^+^IL-17A^+^ T cells was determined by flow cytometry (G). (H-I) The naïve CD4^+^ T cells were transfected with GLS1 plasmid, and then treated with anti-CD3/CD28, Th17-skewing cytokines, ROSI (30 µM) or PIO (30 µM) for 48 h. The concentration of intracellular GSH was detected by using a commercial kit (H), and the level of ROS was determined by flow cytometry (I). (J-K) The naïve CD4^+^ T cells were treated with anti-CD3/CD28 in the presence or absence of Th17-skewing cytokines, NAC (1 mM), ROSI (30 µM) as well as PIO (30 µM) for 72 h. The mRNA expression of RORγt was detected by Q-PCR (J), and the frequency of CD4^+^IL-17A^+^ T cells was determined by flow cytometry (K). Data were presented as the means ± S.E.M. of three independent experiments. ^#^*P* < 0.05, ^##^*P* < 0.01 vs. Control group; ^*^P < 0.05, ^**^P < 0.01 vs. Th17 group (Model group); ^$^*P* < 0.05, ^$$^*P* < 0.01 vs. ROSI group; ^&^*P* < 0.05, ^&&^*P* < 0.01 vs. PIO group.

The results showed that ROSI (10, 30 μM) and PIO (10, 30 μM) significantly down-regulated the concentration of intracellular GSH and up-regulated the level of ROS (Figure 6A-B). However, the mRNA expression levels of GCLC, GCLM and GS were not altered by ROSI (3, 10, 30 μM) and PIO (3, 10, 30 μM) (Figure 6C), implying that PPARγ agonists have an indirect effect on GSH de novo synthesis. As revealed in Figure 6D-G, the effects of PPARγ agonists on the GSH/ROS bypath, the mRNA expression of RORγt and the frequency of CD4^+^IL-17A^+^ T cells were obviously reduced by exogenous glutamate. Moreover, the effects of PPARγ agonists on the concentration of GSH and the level of ROS were abolished by the GLS1 plasmid (Figure 6H-I), while replenishing GSH by using N-acetyl-L-cysteine (NAC) reversed the inhibitory effects of PPARγ agonists on the mRNA expression of RORγt and Th17 differentiation (Figure 6J-K). These data suggest that the GSH/ROS axis is the link between the PPARγ agonists-reduced level of GLS1 and the mRNA expression of RORγt.

### The regulation of GLS1-mediated glutaminolysis, subsequent signals and Th17 responses by PPARγ agonists is dependent on PPARγ

**Figure 7:**
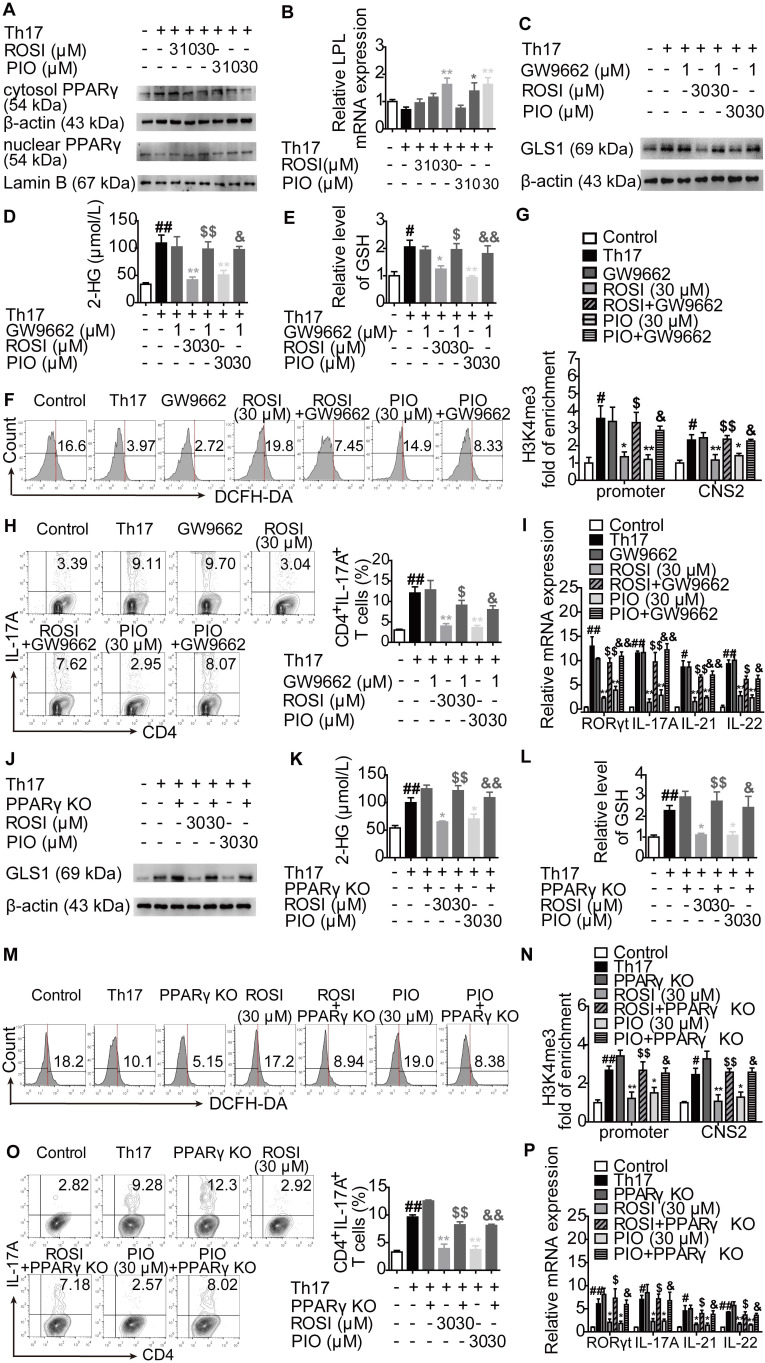
** The regulation of PPARγ agonists on GLS1-mediated glutaminolysis, subsequent signals and Th17 responses exerts a PPARγ-dependent feature.** (A-B) The naïve CD4^+^ T cells were treated with anti-CD3/CD28 in the presence or absence of Th17-skewing cytokines, rosiglitazone (ROSI; 3, 10, 30 µM) and pioglitazone (PIO; 3, 10, 30 µM) for 48 h. The protein level of PPARγ in cytosol or nuclear was analyzed by western blotting assay (A), and the mRNA expression of LPL was examined by Q-PCR (B). (C-I) The naïve CD4^+^ T cells were treated with anti-CD3/CD28 in the presence or absence of Th17-skewing cytokines, GW9662 (1 µM), ROSI (30 µM) as well as PIO (30 µM). After 48 h, the protein expression of GLS1 was analyzed by western blotting assay (C), the concentrations of intracellular 2-HG (D) and GSH (E) were detected by using commercial kits, and the level of ROS was determined by flow cytometry (F). After 72 h, the enrichment of H3K4me3 in promoter and CNS2 region of *il-17* gene was analyzed by ChIP (G), the frequency of CD4^+^IL-17A^+^ T cells was determined by flow cytometry (H), and the mRNA expression levels of RORγt, IL-17A, IL-21 as well as IL-22 were examined by Q-PCR (I). (J-P) The naïve CD4^+^ T cells were transfected with PPARγ CRISPR/Cas9 KO plasmid, followed by treatment of anti-CD3/CD28, Th17-skewing cytokines, ROSI (30 µM) or PIO (30 µM). After 48 h, the protein expression of GLS1 was analyzed by western blotting assay (J), the concentrations of intracellular 2-HG (K) and GSH (L) were detected by using commercial kits, and the level of ROS was determined by flow cytometry (M). After 72 h, the enrichment of H3K4me3 in promoter and CNS2 region of *il-17* gene was analyzed by ChIP (N), the frequency of CD4^+^IL-17A^+^ T cells was determined by flow cytometry (O), and the mRNA expression levels of RORγt, IL-17A, IL-21 as well as IL-22 were examined by Q-PCR (P). Data were presented as the means ± S.E.M. of three independent experiments. ^#^*P* < 0.05, ^##^*P* < 0.01 vs. Control group or the group without any treatment; ^*^P < 0.05, ^**^P < 0.01 vs. Th17 group (Model group); ^$^*P* < 0.05, ^$$^*P* < 0.01 vs. ROSI group; ^&^*P* < 0.05, ^&&^*P* < 0.01 vs. PIO group.

Both ROSI and PIO are classical PPARγ agonists, but they can function independent of PPARγ. To determine whether ROSI and PIO acted in a PPARγ-dependent manner under Th17-skewing conditions, the nuclear translocation and transcriptional activity of PPARγ were first examined. As shown in Figure 7A, the nuclear level of PPARγ was increased when cells were treated with ROSI and PIO, while the cytosolic level was decreased. The transcriptional activity of PPARγ indicated by the mRNA level of its target gene LPL was also up-regulated by ROSI (30 μM) and PIO (10, 30 μM) (Figure 7B), suggesting that they did activate PPARγ.

The involvement of PPARγ in ROSI- and PIO-regulated GLS1/2-HG/H3K4me3 and GLS1/GSH/ROS signals was further detected by combination treatment with the PPARγ antagonist GW9662. The results showed that the down-regulation of ROSI (30 μM) and PIO (30 μM) on the expression of GLS1, the intracellular level of 2-HG, the levels of H3K4me3 in the promoter and CNS2 region of the *il-17* locus, and the intracellular level of GSH was rescued by GW9662 (1 μM), while the up-regulation of the intracellular level of ROS was abolished (Figure 7C-G). In a more comprehensive experiment, the CRISPR/Cas9 KO plasmid for knocking out PPARγ was used, and similar results were obtained, as indicated by Figure 7J-N. Moreover, Figure 7H-I, O-P revealed that, by interfering with PPARγ, the inhibitory effects of ROSI (30 μM) and PIO (30 μM) on the frequency of CD4^+^IL-17A^+^ T cells and the mRNA expression levels of RORγt, IL-17A, IL-21 and IL-22 almost disappeared. These results indicate the exact role PPARγ plays in ROSI- and PIO-regulated GLS1/2-HG/H3K4me3 and GSH/ROS signaling as well as Th17 responses.

### PPARγ agonists ameliorate Th17-related diseases by targeting GLS1-mediated glutaminolysis/2-HG/H3K4me3 and GSH/ROS signals

**Figure 8:**
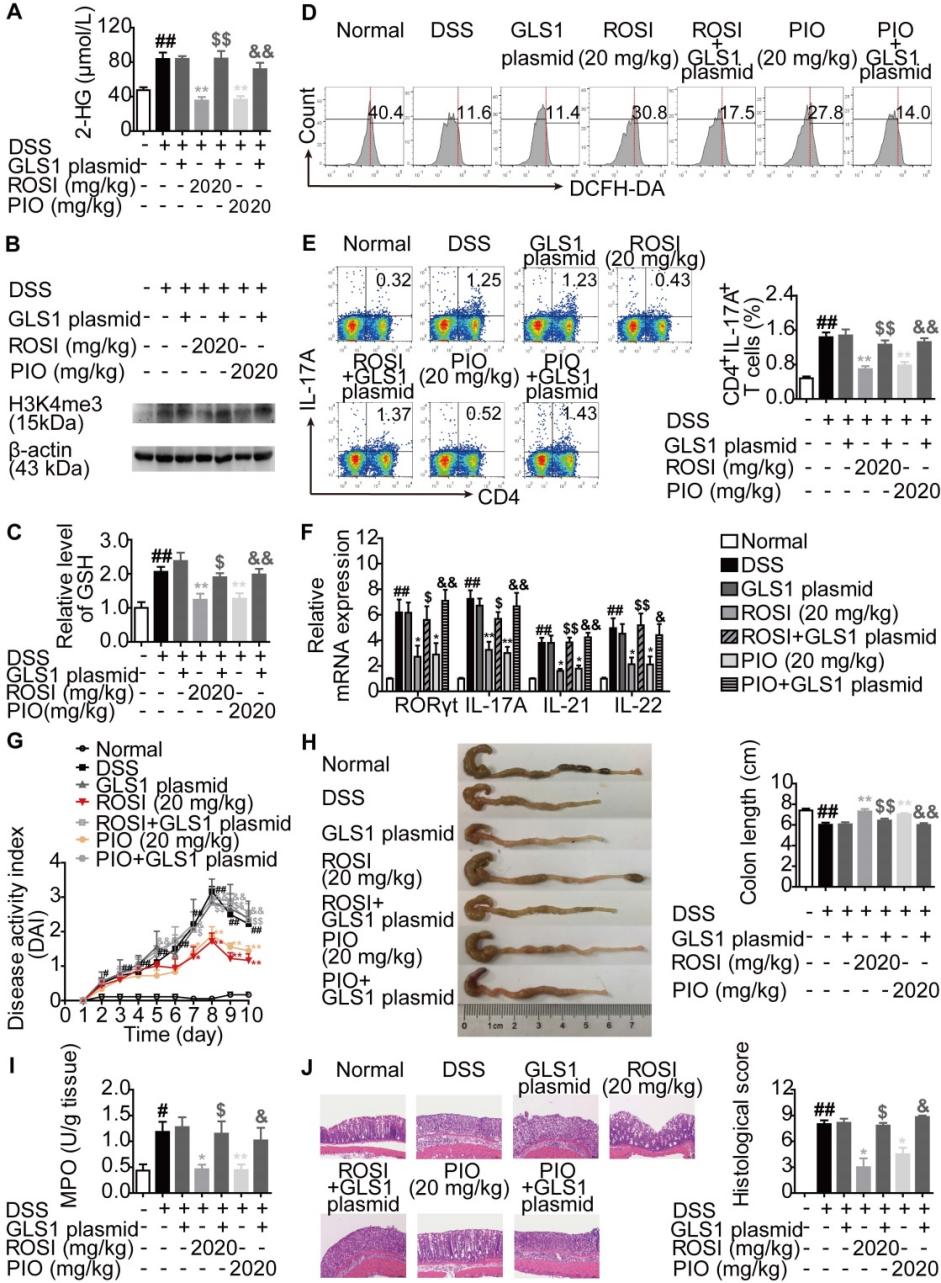
** PPARγ agonists restrict colitis in mice *via* down-regulating GLS1-mediated glutaminolysis.** The colitis model in mice was induced by dextran sulfate sodium (DSS). GLS1 plasmid (10 µg/mouse; p.r.), rosiglitazone (ROSI; 20 mg/kg; i.g.) and pioglitazone (PIO; 20 mg/kg; i.g.) were administered. (A) The level of 2-HG in colons was determined by using a commercial kit. (B) The level of H3K4me3 in colons was analyzed by western blotting assay. (C) The level of GSH in lymphocytes of colonic lamina proprias was detected by using a commercial kit. (D) The level of ROS in lymphocytes of colonic lamina proprias was analyzed by flow cytometry. (E) The frequency of CD4^+^IL-17A^+^ T cells in mesenteric lymph nodes was determined by flow cytometry. (F) The relative mRNA expression levels of RORγt, IL-17A, IL-21 and IL-22 in colons were examined by Q-PCR. (G) The DAI score was calculated. (H) The colon length was analyzed. (I) The MPO activity was determined by using a commercial kit. (J) The histological changes in colons were analyzed by H&E staining (×200). Data were presented as the means ± S.E.M. (n = 6 in each group). ^#^*P* < 0.05, ^##^*P* < 0.01 vs. Normal group; ^*^P < 0.05, ^**^P < 0.01 vs. DSS group (Model group); ^$^*P* < 0.05, ^$$^*P* < 0.01 vs. ROSI group; ^&^*P* < 0.05, ^&&^*P* < 0.01 vs. PIO group.

The mechanisms by which PPARγ agonists restricted Th17 responses were further confirmed *in vivo*. First, colitis in mice was induced by dextran sulfate sodium (DSS), and ROSI (20 mg/kg) as well as PIO (20 mg/kg) were administered in combination with the GLS1 plasmid. The results showed that the two PPARγ agonists significantly reduced the levels of 2-HG and H3K4me3 in colons of mice with colitis, regulated the levels of GSH and ROS in lymphocytes of colonic lamina propria, and the effects were weakened by GLS1 overexpression (Figure 8A-D). The down-regulated frequency of Th17 cells in mesenteric lymph nodes (MLNs) and the decreased mRNA expression levels of RORγt, IL-17A, IL-21 and IL-22 in colons caused by ROSI and PIO were also rescued by GLS1 overexpression (Figure 8E-F). In addition, the administration of the GLS1 plasmid markedly weakened the amelioration of PPARγ agonists on the disease activity index (DAI) scores, shortening of colon length, MPO activity, and histological alterations in the colons of mice treated with DSS (Figure 8G-J).

**Figure 9:**
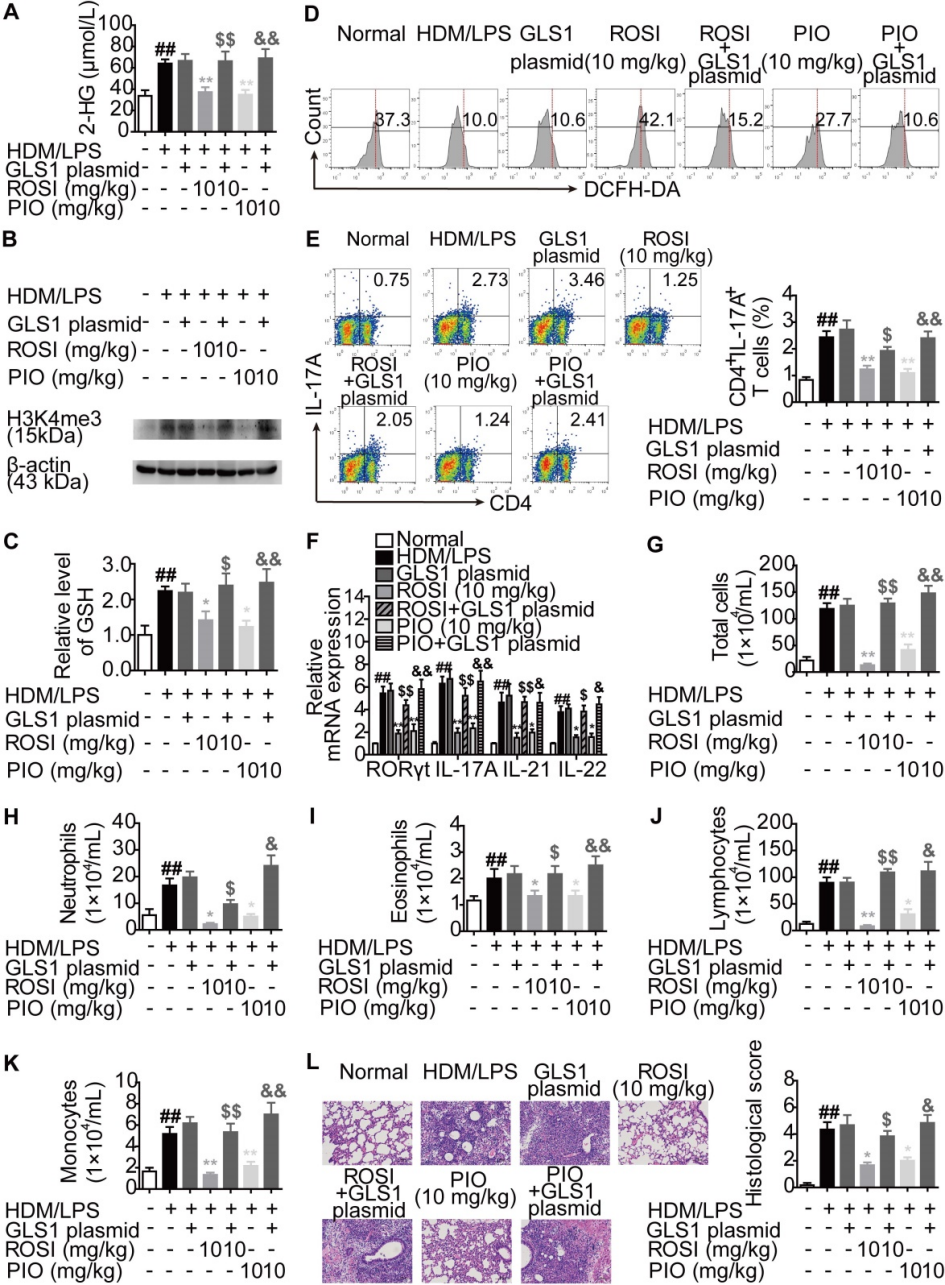
** PPARγ agonists inhibit asthma in mice *via* down-regulating GLS1-mediated glutaminolysis.** The neutrophilic asthma model in mice was induced by house dust mite (HDM)/lipopolysaccharide (LPS). GLS1 plasmid (10 µg/mouse; i.n.), rosiglitazone (ROSI; 10 mg/kg; i.g.) and pioglitazone (PIO; 10 mg/kg; i.g.) were administered. (A) The level of 2-HG in lungs was determined by using a commercial kit. (B) The level of H3K4me3 in lungs was analyzed by western blotting assay. (C) The level of GSH in lymphocytes of lungs was detected by using a commercial kit. (D) The level of ROS in lymphocytes of lungs was analyzed by flow cytometry. (E) The frequency of CD4^+^IL-17A^+^ T cells in hilar lymph nodes was determined by flow cytometry. (F) The relative mRNA expression levels of RORγt, IL-17A, IL-21 and IL-22 in lungs were examined by Q-PCR. (G-L) The numbers of total cells (G), neutrophils (H), eosinophils (I), lymphocytes (J) and monocytes (K) in bronchoalveolar lavage fluids (BALFs) were analyzed. (L) The histological changes in lungs were analyzed by H&E staining (×200). Data were presented as the means ± S.E.M. (n = 6 in each group). ^##^*P* < 0.01 vs. Normal group; ^*^P < 0.05, ^**^P < 0.01 vs. HDM/LPS group (Model group); ^$^*P* < 0.05, ^$$^*P* < 0.01 vs. ROSI group; ^&^*P* < 0.05, ^&&^*P* < 0.01 vs. PIO group.

Moreover, an HDM/lipopolysaccharide (LPS)-induced asthma model was established in mice, and the impact of GLS1 overexpression on the biological activities of ROSI (10 mg/kg) and PIO (10 mg/kg) was examined. The results were similar to those obtained from the colitis model, as GLS1 overexpression effectively dampened the restriction of PPARγ agonists on the level of 2-HG as well as H3K4me3 in the lungs, and the regulation of the levels of GSH and ROS in lymphocytes in the lungs (Figure 9A-D). Consistently, the effects of ROSI (10 mg/kg) and PIO (10 mg/kg) on Th17 responses, the numbers of inflammatory cells in bronchoalveolar lavage fluids (BALFs), and the histological changes in lungs in mice with asthma were also weakened by the GLS1 plasmid (Figure 9E-L). These data imply that PPARγ agonists can ameliorate Th17 cell-related colitis and asthma by inhibiting GLS1-mediated glutaminolysis/2-HG/H3K4me3 and GSH/ROS signals.

## Discussion

Th17 cells have long been considered pro-inflammatory cells despite their capacity for immune defense. When stimulated by TGF-β, IL-6 and IL-23, activated Th0 cells acquire the ability to differentiate into pathogenic Th17 cells and produce related cytokines, with IL-17A being the best-defined functional effector 26. Considering the pathological effects, intensive research endeavors have been invested in determining the molecular target for regulating the Th17/IL-17A axis. An appealing concept is the transcription factor PPARγ, whose pivotal role in controlling Th17 responses has been discovered by using agonists as well as methods to intervene in gene expression 8. The classical PPARγ agonists thiazolidinediones can activate PPARγ in a ligand-dependent manner. Among them, both ROSI and PIO have been proved to reduce the number of CD4^+^IL-17A^+^ T cells in the differentiation programs *in vitro* 27,28. Therefore, to be more comprehensive, we attempted to illustrate the mechanisms underlying how PPARγ could control Th17 responses by using these two tool drugs.

Glycolysis, glutaminolysis and fatty acid metabolism are three main metabolic pathways in cells. Similar to other quiescent cells, naïve CD4^+^ T cells preferentially use fatty acid oxidation to meet energetic demands 29. However, upon T cell receptor (TCR) ligation and co-stimulation, they undergo a metabolic switch towards glycolysis and glutaminolysis to fuel the activation, proliferation, and production of cytokines 30. Under Th17-skewing conditions, further increased glutaminolysis has been observed, and the inhibition of multiple involved enzymes can result in defective Th17 responses 15,30. In the case of glycolysis, although its convincing engagement in Th17 responses is evidenced by the blockage of HIF-1α, an essential transcription factor related to this pathway 31, the role it played in Th17 responses remains to be elucidated, as several lines of studies indicate no stronger glycolysis in differentiating Th17 cells compared with Th0 cells 15,32,33. A possible explanation is the crosstalk between the multiple metabolic pathways. For example, a newly identified stronger hexosamine biosynthesis resulting from limited glycolysis contributes to the restriction of Th17 responses by favoring differentiation into Treg cells 34. In this study, we found stronger glutaminolysis but not glycolysis under Th17-skewing conditions, and ROSI and PIO could not constrain glycolysis but possessed a significant inhibitory effect on glutaminolysis. The effect of glutamine on Th17 responses was also counteracted by ROSI and PIO, implying that glutaminolysis lay at the nexus of the PPARγ agonists-mediated impact on Th17 cells. Moreover, the inhibition of PPARγ agonists on Th17 cells after inhibiting glutaminolysis was proved to be a direct action, as the possibility that PPARγ agonists promoted the shift of Th17 cells towards Treg cells was ruled out.

Glutaminolysis is a kind of amino acid metabolism starting from the uptake of extracellular glutamine *via* transporters such as SLC1A5 and SLC38A1 17,18. Then, the conversion of intracellular glutamine to α-KG is catalyzed consecutively by GLS, GLUD or transaminases 17,18. Of note, α-KG is an intermediate of the tricarboxylic acid (TCA) cycle, which can be further converted to succinate by α-KG dehydrogenase (KGDH) 23. Additionally, α-KG is a source of 2-HG, whose generation is mediated by IDH 19. Considering that glutaminolysis can be divided by the processes of uptake and conversion, we separately detected the effects of ROSI and PIO on the two processes and determined that the transformation of glutamine to glutamate was restricted in accordance with the diminished expression of GLS1, the rate-limiting enzyme of this step. The involvement of GLS1 in PPARγ agonists-mediated inhibition of Th17 differentiation was further confirmed by GLS1 overexpression. In addition, the glutamine metabolite 2-HG directly functioned in indicating the fate of Th17 cells, and the effects of ROSI and PIO on IDH1/2 were excluded in this study, revealing that PPARγ agonists ablated Th17 differentiation by regulating the GLS1/2-HG axis. Interestingly, we did not find a link between PPARγ agonists-regulated 2-HG level and RORγt expression, although the relationship between glutaminolysis and RORγt expression was confirmed by GLS1 overexpression and glutamine deprivation studies. Thus, by inhibiting the GLS1/2-HG axis, PPARγ agonists decreased IL-17A expression independent of RORγt expression, and additional signals were regulated subsequent to the reduced protein level of GLS1, which led to limited RORγt expression.

Histone methylation is a covalent modification that plays important roles in chromatin remodeling and gene transcription 35. Generally, the modifications of H3K9me3 and H3K27me3 are associated with gene silencing, while H3K4me3 is related to active transcription 35. By regulating the enrichment of methyl groups at the specific sites of histones, histone methyltransferases and demethylases are engaged in controlling gene expression as well as relevant physiological and pathological processes 33. Among the demethylases, JMJD is a special class whose activity can be antagonized by 2-HG 23. It is noteworthy that a wealth of data suggests a close relationship between histone methylation and Th17 responses, as the level of H3K4me3, which can be regulated by KDM5 (a member of the JMJD class), is up-regulated in the *il-17* gene locus of CD4^+^ T cells during Th17 differentiation 21,22. In addition, PPARγ, when activated by a prostaglandin I2 analog, can inhibit the H3K4me3 modification in the gene promoter region and subsequent expression of IP-10 in LPS-stimulated THP-1 cells, indicating the effect of PPARγ on histone methylation 36. Therefore, we explored how PPARγ agonists-reduced level of 2-HG resulted in limited IL-17A expression as well as the Th17 responses in view of histone methylation and found that PPARγ agonists could down-regulate the H3K4me3 level in the promoter and CNS2 region of the *il-17* gene, which subsequently abolished the generation of Th17 cells.

According to the above-mentioned results, we could conclude that PPARγ agonists decreased IL-17A expression by regulating GLS1/2-HG/H3K4me3 signals, which was independent of RORγt expression. However, the mechanisms by which PPARγ agonists limited RORγt expression were still unclear. Recently, GSH de novo synthesis has been confirmed to direct T cell differentiation by controlling redox homeostasis 24. Genetic ablation of enzymes in GSH de novo synthesis leads to the augmentation of ROS and limitation of Th17 differentiation, and intervention in ROS can result in the disturbance of RORγt expression with unclear mechanisms 24, 37, 38. However, signal transducers and activator of transcription 3 (STAT3) may be a causal link between ROS and RORγt, as the oxidative status can dampen its activation, which discourages the transcription of RORγt 37. Of note, in the process of de novo synthesis, glutamate is the raw material to form dipeptide γ-glutamylcysteine (γ-GC), the first and rate-limiting step catalyzed by GCLC and GCLM, and γ-GC is ligated with glycine by GS to form GSH 24. This means that heightened glutaminolysis during the progression of Th17 differentiation provides glutamate to support GSH synthesis, and the GSH/ROS axis can be considered a bypath of glutaminolysis. Evidence has confirmed this theory, as NAC weakens the inhibition of CB839, a GLS1 inhibitor, on RORγt expression and the frequency of IL-17^+^ cells 25. We therefore determined whether the effect of PPARγ agonists on RORγt expression was related to GLS1/GSH/ROS signaling, and the results showed the actual connection between them.

Although both ROSI and PIO are classical PPARγ agonists, several lines of evidence suggest that they can function independent of PPARγ. For example, ROSI dampens the expression of pigment epithelium-derived factor (PEDF), a driver of insulin resistance, in hepatocytes and adipocytes by activating AMP-activated protein kinase (AMPK) instead of PPARγ 39. PIO induces the apoptosis of HEp-2 and HSC-3 human cancer cells through inhibition of STAT3 and enhancement of apoptosis-inducing factor (AIF) expression in a PPARγ-independent manner 40. In our study, we excluded this possibility, as the inhibitory effects of ROSI and PIO on GLS1-mediated metabolism and subsequent Th17 responses were rescued by using the PPARγ antagonist GW9662 or knocking out the PPARγ gene.

*In vivo*, Th17 cells are largely distributed to barrier sites, such as the intestine and lung 10. Consistent with this distribution, a wide array of studies have indicated that colitis and asthma are two kinds of Th17 cell-related diseases. In the colons of patients and mice with colitis, Th17 responses are much stronger than those in heathy controls, while the frequencies of Th17 cells together with the levels of IL-17A in the lungs and BALFs of mice with asthma are also up-regulated 41-44. Moreover, interfering with Th17 responses by neutralizing IL-17A attenuates the progression of colitis and asthma 45,4. Therefore, we determined the effects of PPARγ agonists on mice with colitis or asthma and further confirmed the critical effect and mechanism of GLS1-mediated metabolism in PPARγ-mediated function, as GLS1 overexpression rescued the* in vivo* inhibitory effects of ROSI and PIO on 2-HG/H3K4me3 and GSH/ROS signals, Th17 responses and disease features.

In summary, PPARγ agonists abolish Th17 responses through inhibiting glutaminolysis, thereby ameliorating Th17 cell-related inflammation and autoimmune diseases. The precise mechanisms can be summarized as follows: 1) abrogating GLS1, reducing the level of 2-HG, and regulating KDM5-specific H3K4me3 modifications in the promoter and CNS2 region of the *il-17* gene locus; and 2) abrogating GLS1, reducing the level of GSH, increasing the level of ROS, and down-regulating the expression of RORγt.

## Materials and Methods

### Reagents

ROSI (C_18_H_19_N_3_O_3_S, MW: 357.43; purity > 98%) and PIO (C_19_H_20_N_2_O_3_S, MW: 356.44; purity > 98%) were purchased from TargetMol (Shanghai, China) and CSNpharm, Inc. (Chicago, USA) respectively. Mouse CD4^+^CD62L^+^ T-cell isolation kit was purchased from Miltenyi Biotech (Cologne, Germany), and rhTGF-β1, rmIL-6, rmIL-23 were purchased from PeproTech (Madison, USA). Purified anti-mouse CD3e/CD28 mAbs, FITC-anti-CD4, APC-anti-CD25 and PE-anti-Foxp3 were purchased from eBioscience (San Diego, USA). APC-anti-IL-17A was purchased from BioLegend (San Diego, USA). Fixation & permeabilization kit, PMA/Ionomycin mixture and BFA/Monensin mixture were purchased from MultiSciences Biotech (Hangzhou, China). Antibodies against GLUD1, GOT1, GPT2, IDH1, IDH2 and Foxp3 were purchased from Sangon Biotech (Shanghai, China). Antibody against GLS1 was purchased from ABclonal (Woburn, USA). Antibodies against PPARγ was purchased from Wanleibio (Shenyang, Liaoning). Antibody against β-actin was purchased from Bioworld Technology, Inc. (Atlanta, USA). Mouse glutamine, glutamate, succinate, α-KG, 2-HG, lactate and GSH detection assay kit were purchased from Jiancheng Bio-engineering Institute (Nanjing, China). Cell-permeable octyl-α-KG and octyl-2-HG were purchased from Cayman (Ann Arbor, USA). Cell-permeable dimethyl-succinate was purchased from Aladdin Bio-Chem Technology Co., Ltd. (Shanghai, China). Chip grade anti-H3K4me3 antibody was purchased from Affinity Biosciences (Cincinnati, USA). Chip assay kit and DCFH-DA were purchased from Beyotime Biotechnology (Shanghai, China). ML324, CPI-455 and GSK-J4 were purchased from TargetMol (Shanghai, China). HiScript™ reverse transcriptase system and AceQ™ qPCR SYBR® Green Master Mix were purchased from Vazyme Biotech Co., Ltd. (Piscataway, USA). Lipofectamine 2000 and TRIzol were purchased from Invitrogen (Carlsbad, USA). DSS (molecular weight: 36-50 kDa) was purchased from MP Biomedical (Solon, USA). LPS was purchased from Sigma-Aldrich (St. Louis, USA). HDM was purchased from Greer Laboratories (Lenoir, USA). Entranster *in vivo* transfection reagent was purchased from Engree Biosystems Co. (Beijing, China).

### Animals

Female C57BL/6 mice weighing 18-22 g were purchased from the Comparative Medicine Centre of Yangzhou University (Yangzhou, China). The animal experiments were conducted with the approval of the Animal Ethics Committee of China Pharmaceutical University and complied with the National Institute of Health guidelines on the ethical use of animals. All animals were housed under a 12 h light/dark cycle (21 ± 2 °C) and were allowed ad libitum access to a diet of standard laboratory chow and water.

### Induction of UC and treatments

Mice were randomly divided into 7 groups with 6 mice in each: normal group, model group, GLS1 plasmid group, ROSI group, ROSI+GLS1 plasmid group, PIO group and PIO+GLS1 plasmid group. Except for the normal group, mice were fed with 2.5% (w/v) DSS dissolved in sterile distilled water for 7 days, followed by sterile distilled water alone for another 3 days. ROSI (20 mg/kg) and PIO (20 mg/kg) were orally administered once a day for a total of 10 days. The GLS1 plasmid was mixed with an equal volume of Entranster *in vivo* transfection reagent, and was rectally administered daily throughout the experiment.

Body weight, diarrhea and hematochezia were measured every day. The DAI scores were accounted by the mean values of the following: (a) weight loss (0=none; 1=1-5%; 2=5-10%; 3=10-15%; 4=over 15%); (b) diarrhea scores (0=normal; 2=loose stools; 4=diarrhea); and (c) blood stool scores (0=normal; 2=hemoccult; 4=gross bleeding).

On day 10, the colons of mice were collected and fixed in 10% formalin for histological examination. The histological scores were graded based on a scoring system that included the following 46: (a) severity of inflammation: 0=none; 1=slight; 2=moderate; 3=severe; (b) sites of inflammation: 0=none; 1=mucosa; 2=mucosa and submucosa; 3=transmural; and (c) crypt lesions: 0=none; 1=basal 1/3 damaged; 2=basal 2/3 damaged; 3=only surface epithelium intact; 4=entire crypt and epithelium lost. A maximal score of 10 was assessed by summing up the three evaluations.

### Establishment of neutrophilic asthma and treatments

To establish the neutrophilic asthma model, repeated HDM extract plus LPS intranasal challenges were performed in mice 47,48. Mice were randomly divided into normal, model, GLS1 plasmid, ROSI, ROSI+GLS1 plasmid, PIO and PIO+GLS1 plasmid groups (n = 6 in each group). On day 0, mice were intranasally sensitized with either sterile saline (40 μL) alone or a combination of HDM (20 μg) and LPS (5 μg). Then, the mice were further challenged with the combination of HDM (10 μg) and LPS (5 μg) daily from day 7 to 11. ROSI (10 mg/kg) and PIO (10 mg/kg) were orally administered once a day from day 7 for 9 consecutive days. The GLS1 plasmid was mixed with an equal volume of Entranster *in vivo* transfection reagent, and intranasally administered every other day throughout the experiment.

On day 15, mice were harvested, and the BALF was collected. In addition, the lower lobe of the right lung was fixed with 10% formalin, and processed for hematoxylin-eosin (H&E) staining. The following parameters were scored: peribronchial inflammation, interstitial inflammation, endothelialitis, edema and pleuritis. Each parameter was graded on a scale of 0 to 4 (0: absent, 1: mild, 2: moderate, 3: severe, 4: very severe). The total histological scores were expressed as the sum of the score for all parameters 49.

### Cell culture and differentiation

Naïve CD4^+^ T cells isolated from MLNs of mice were purified with magnetic beads according to the manufacturer's instructions for the CD4^+^CD62L^+^ T Cell Isolation Kit (Miltenyi Biotech, Cologne, Germany). The cells were maintained in RPMI 1640 (Gibco, Carlsbad, CA, USA) supplemented with 10% fetal bovine serum (FBS) under a humidified 5% (v/v) CO_2_ atmosphere at 37 °C.

For the induction of Th17 cells, naïve CD4^+^ T cells were treated with plate-bound anti-CD3 (1 μg/mL), anti-CD28 (1 μg/mL), rhTGF-β1 (2 ng/mL), rmIL-6 (40 ng/mL) and rmIL-23 (10 ng/mL) for 72 h. ROSI, PIO, glutamine, α-KG, succinate, 2-HG, CPI-455, glutamate and GW9662 were added at the beginning of Th17 cell induction. The frequency of Th17 cells was detected by flow cytometry, which was performed as follows.

### Intracellular staining and flow cytometry

For Th17 intracellular staining, lymphocytes harvested from *in vitro* culture or tissues were stimulated with a PMA/Ionomycin mixture. Meanwhile, a BFA/Monensin mixture was added to block protein transport. Then, the cells were cultured for 5 h at 37 °C and stained with FITC-anti-CD4 for 30 min at 4 °C. After fixation and permeabilization, they were exposed to APC-anti-IL-17A for 1 h. Finally, the flow cytometric measurements were performed on a FACSCalibur (BD Biosciences, San Jose, CA, USA).

### Cell viability assay

The cells were seeded into a 96-well plate at a density of 1×10^6^ cells/mL and were treated with ROSI (1, 3, 10, 30, 100 μM) or PIO (1, 3, 10, 30, 100 μM) for 72 h. At 4 h before the end of incubation, 20 μL of MTT solution (5 mg/mL dissolved in PBS) was added into each well. Then, the supernatant was discarded, and 150 μL of DMSO was added. The crystals were sufficiently dissolved, and the optical absorbance value was measured at 570 nm. In another case, 10 μL of CCK-8 was added to each well 4 h before the end of incubation, and the optical absorbance value was measured at 450 nm.

### Cell apoptosis detection

Cell apoptosis was detected by flow cytometry after Annexin V-FITC/PI staining. Briefly, cells were washed with phosphate buffer saline (PBS), and re-suspended with 500 µL binding buffer. The suspension was then added a volume of 5 µL Annexin V-FITC and 10 µL PI. After 10 min incubation at room temperature, cells were analyzed on FACS Calibur.

### Q-PCR assay

The total RNAs of cells or tissues were extracted by using TRIzol reagent and HiScript™ reverse transcriptase. Then, the Super Mix was used to reverse transcribe the RNAs into cDNAs. Finally, Q-PCR assay of target genes was performed on the Bio-Rad CFX Connect real-time PCR system (Bio-Rad, USA). The mRNA expression levels of RORγt, IL-17A, IL-21 and IL-22 were normalized to β-actin. In addition, the details of the gene-specific primers (Sangon Biotech, Shanghai, China) were listed in Table 1.

### Western botting assay

The cells or tissues were lysed by using NP40 buffer that containing 1 mM PMSF on ice for 30 min, and were centrifuged at 12 000 rpm for 10 min. Then, supernatants were collected, and the protein lysates were further prepared by adding loading buffer. All the samples were separated by 10% SDS-PAGE, and transferred onto pre-activated polyvinylidene fluoride (PVDF) membranes. The membranes were blocked with 5% nonfat milk for 2 h at room temperature, and probed with primary antibodies overnight at 4 °C. After being washed, the membranes were incubated with secondary antibodies for 2 h at room temperature. The blots were finally visualized by using enhanced chemiluminescent (ECL) reagent.

### Transfection

siFoxp3 (sense: 5'-AAAGGUUGCUGUCUUUCCUGGGUGU -3'; anti-sense: 5'-ACACCCAGGAAAGACAGCAACCUUU-3'); siIDH1 (sense: 5'-GAAUUCAAGUUGAAACAAAUG-3'; anti-sense: 5'-UUUGUUUCAACUUGAAUUCUU-3') and siIDH2 (sense: 5'-GCGACCAGUACAAGGCCACAGAUUU3'; anti-sense: 5'-AAAUCUGUGGCCUUGUACUGGUCGC-3') were synthesized by RiboBio Co. (Guangzhou, China). The GLS1 overexpression plasmid and PPARγ CRISPR/Cas9 KO plasmid were designed and constructed by Genomeditech (Shanghai, China). Transfection was performed by using Lipofectamine 2000 according to the manufacturer's instructions, and the cells were incubated with transfection complexes for 48 or 72 h in the presence or absence of ROSI, PIO as well as α-KG for further investigation.

### ChIP assay

The cells were harvested and incubated with 1% formaldehyde for 10 min at 37 °C for crosslinking. After the reaction was quenched by adding glycine, the cells were washed with PBS containing 1 mM PMSF. The precipitates were resuspended in SDS lysis buffer (including 1 mM PMSF) and subsequently sonicated (amplitude, 40 W; process time, 6 min; ON time, 4.5 s; OFF time, 9 s). Immunoprecipitation was further carried out by adding antibody against H3K4me3 and protein A+G Agarose/Salmon Sperm according to the manufacturer's instructions. Moreover, the protein-DNA complexes were de-crosslinked at 65 °C for 4 h, followed by proteinase K treatment so as to degrade the protein. The acquired DNAs were purified by using a commercial kit (Beyotime Biotech, Nanjing, China), and the enrichment was detected by Q-PCR. The primers used were listed in Table 1.

### Statistical analysis

SPSS software (SPSS, Chicago, IL, USA) was used, and data were presented as the mean ± S.E.M. Student's t-test was performed to compare the mean differences between two groups. One-way ANOVA followed by the LSD test was conducted to compare the mean differences between multiple groups, and in cases where the latter condition was violated, non-parametric Games-Howell post hoc test was used. A value of p less than 0.05 (*p* < 0.05) was accepted as a significant difference.

## Figures and Tables

**Table 1:** Primers used in Q-PCR and ChIP

Primers		Sequence (5'-3')
RORγt (mouse)	Forward	TACCCTACTGAGGACAGG
	Reverse	CCACATTACACTGCTGGCTG
IL-17A (mouse)	Forward	TACCTCAACCGTTCCACGTC
	Reverse	TTTCCCAACCGCATTGACACA
IL-21 (mouse)	Forward	TGGATCCTGAACTTCTATCAGC
	Reverse	CACGAGGTCAATGATGAATGTC
IL-22 (mouse)	Forward	GCAGATAACAACACAGATGTCC
	Reverse	GTCTTCCAGGGTGAAGTTGAG
SLC1A5 (mouse)	Forward	GTTACCGCCATCACCTCCATCAAC
	Reverse	GGAAGGCAGCAGACACCAGATTG
SLC38A1 (mouse)	Forward	GAGCACAGGCGACATTCTCATCC
	Reverse	CATGGCGGCACAGGTGGAAC
GCLC (mouse)	Forward	CTATCTGCCCAATTGTTATGGC
	Reverse	CCTCCCGTGTTCTATCATCTAC
GCLM (mouse)	Forward	CTTGGAGCATTTACAGCCTTAC
	Reverse	GTGAGTCAGTAGCTGTATGTCA
GS (mouse)	Forward	CTGATGCTAGAGAGATCTCGTG
	Reverse	TTCACCCATGTCCAGTGAATAG
*il-17* promoter (mouse)	Forward	GCCTTTGTGATTGTTTCTTGCAG
	Reverse	CCTTGCCCAAAGAAACCCTCTC
*il-17* CNS1 (mouse)	Forward	GGAAGGTGCATGTGGCTGACTT
	Reverse	AATGTGCCAGTCCCTTGGATGA
*il-17* CNS2 (mouse)	Forward	GCCTCCCATGTGGTCATTAT
	Reverse	AGGCTCCTTCTCCATTGGTT
*il-17* CNS3 (mouse)	Forward	TTTGCTCATGCCCATATGTC
	Reverse	TGAACCAACTTTCCCCACTC
*il-17* CNS4 (mouse)	Forward	CTCAAATCCGTGTGCCTTCT
	Reverse	CATCTTGAAGCTGAGGCTGA

## Abbreviations

BALF: bronchoalveolar lavage fluid
DAI: Disease activity index
DSS: dextran sulfate sodium
GDH/GLUD: glutamate dehydrogenase
GLS1: glutaminase 1
GOT: glutamic oxaloacetic transaminase
GPT: glutamic pyruvic transaminase
GSH: glutathione
HDM: house dust mite
IDH: isocitrate dehydrogenase
JMJD: JmjC domain-containing demethylases
KGDH: α-ketoglutarate dehydrogenase
LPS: lipopolysaccharide
Pioglitazone: PIO
PPARγ: peroxisome proliferator-activated receptor gamma
PTEN: phosphatase and tensin homologue deleted on chromosome ten
ROS: reactive oxygen species
Rosiglitazone: ROSI
RXRs: retinoid X receptors
α-KG: α-ketoglutarate
2-HG: 2-hydroxyglutarate
