# Supplementary material for: The role of GLS1-mediated glutaminolysis/2-HG/H3K4me3 and GSH/ROS signals in Th17 responses counteracted by PPARγ agonists
Source: Theranostics. 2021 Mar 4;11(9):4531–48. doi: 10.7150/thno.54803 (PMC7977454; doi:10.7150/thno.54803)
Supplement: Supplementary file 1 — Supplementary figures. [file thnov11p4531s1.pdf]

Supplementary Materials

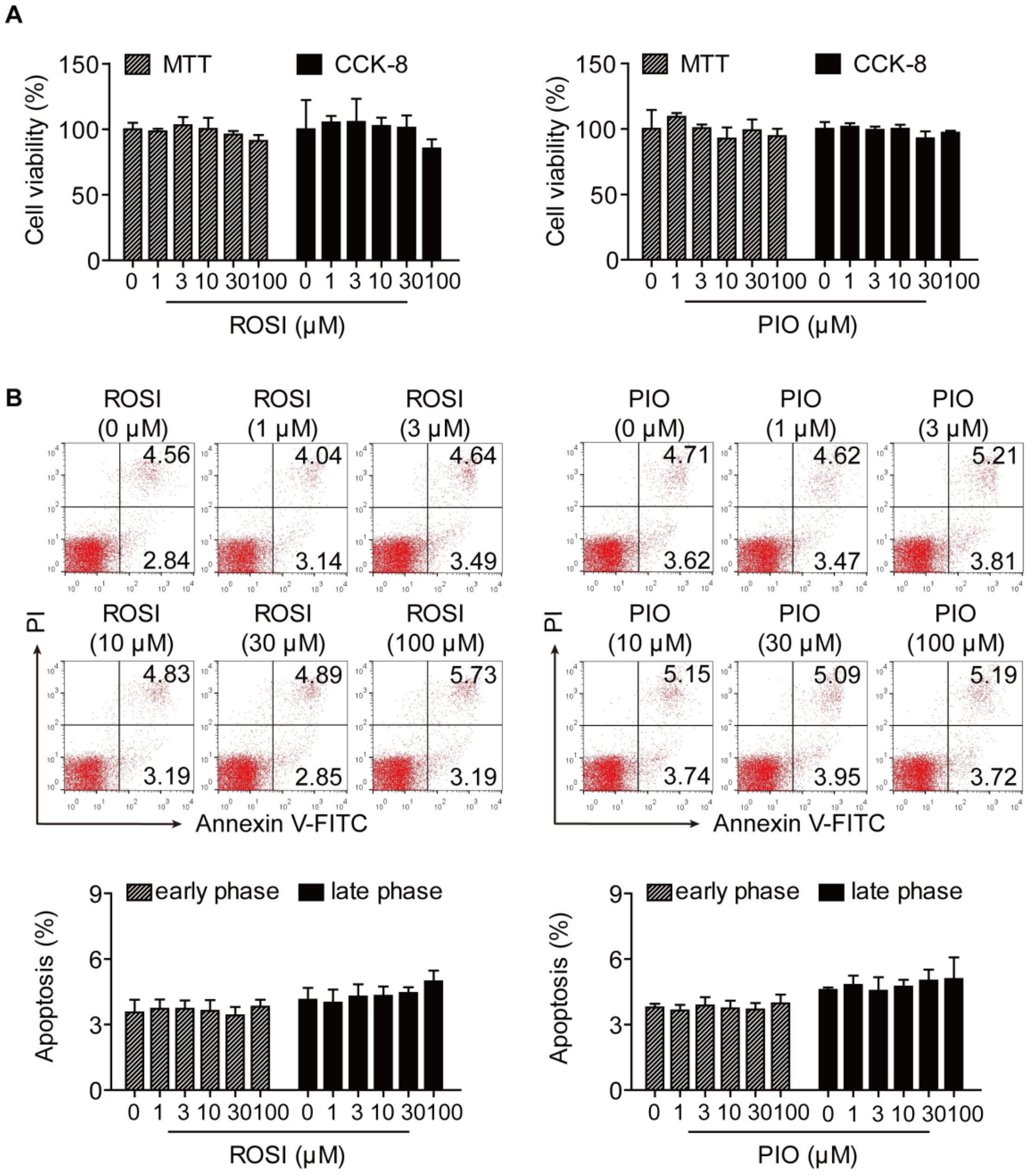

Figure S1 Cellular toxicity of rosiglitazone and pioglitazone. (A-B) Cells isolated from mesenteric lymph nodes were cultured with rosiglitazone (ROSI; 0, 1, 3, 10, 30, 100 μM) and pioglitazone (PIO; 0, 1, 3, 10, 30, 100 μM) for 72 h. The cell viability was analyzed by MTT and CCK-8 assay (A), and the cell apoptosis was examined by Annexin V-FITC/PI assay (B). Data were presented as the means ± S.E.M. of three independent experiments.

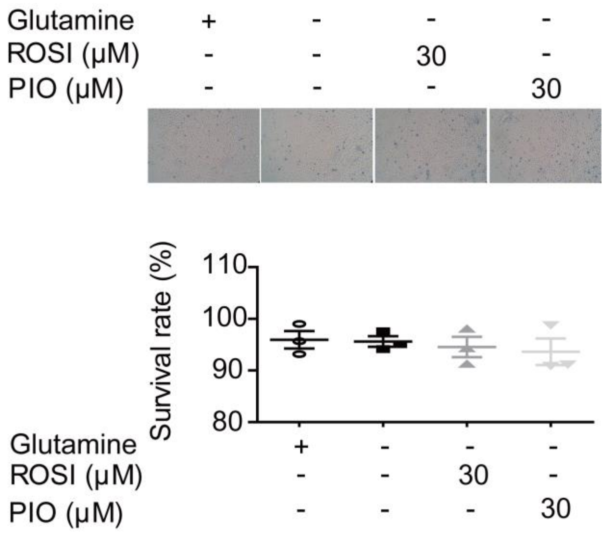

Figure S2 Cellular toxicity of glutamine deprivation. Cells isolated from mesenteric lymph nodes were cultured with or without glutamine (2 mM), rosiglitazone (ROSI; 30 μM) as well as pioglitazone (PIO; 30 μM) for 72 h. The cell survival rate was analyzed by trypan blue exclusion assay. Data were presented as the means ± S.E.M. of three independent experiments.
